# Prevalence of *Mycoplasma genitalium* and Co-Infections with *Chlamydia trachomatis* and *Neisseria gonorrhoeae* Among Japanese Women: A Cross-Sectional Study

**DOI:** 10.3390/idr18020035

**Published:** 2026-04-13

**Authors:** Hiroshige Mikamo, Yuka Yamagishi, Daisuke Sakanashi

**Affiliations:** 1 Department of Clinical Infectious Diseases, Asahi University Hospital, Gifu 500-8856, Japan; saka74d@aichi-med-u.ac.jp; 2 Department of Clinical Infectious Diseases, Aichi Medical University, Nagakute 480-1195, Japan; y.yamagishi@mac.com; 3 Department of Clinical Infectious Diseases, Kochi Medical School, Kochi University, Kochi 783-8505, Japan

**Keywords:** *Mycoplasma genitalium*, sexually transmitted infection, prevalence, *Chlamydia trachomatis*, *Neisseria gonorrhoeae*, Japanese women, pregnancy

## Abstract

**Background/Objectives**: *Mycoplasma genitalium* is an emerging cause of sexually transmitted infections (STIs) and is increasingly recognized for its association with cervicitis and pelvic inflammatory disease. However, prevalence data in specific Japanese subpopulations, particularly comparing pregnant and non-pregnant women, remains limited. This study aimed to determine the prevalence of *M. genitalium* and its co-infection rates with *Chlamydia trachomatis* and *Neisseria gonorrhoeae* among Japanese women. **Methods**: A cross-sectional study was conducted using vaginal swab specimens collected between April 2021 and November 2022 from patients visiting two clinics in Gifu, Japan. The study population comprised 2138 non-pregnant women presenting with urogenital symptoms or sexual contact history, and 236 pregnant women undergoing routine antenatal screening. Detection was performed using real-time polymerase chain reaction assays on the cobas^®^ 8800 system (Roche Diagnostics). **Results**: Among non-pregnant women, the overall prevalence was 3.8% (82/2138) for *M. genitalium*, 3.4% (72/2138) for *C. trachomatis*, and 0.4% (9/2138) for *N. gonorrhoeae*. Co-infection rates were low; *M. genitalium* and *C. trachomatis* co-infection was observed in 0.2% of cases. Among pregnant women, the prevalence was 3.8% (9/236) for both *M. genitalium* and *C. trachomatis*, and 0.4% (1/236) for *N. gonorrhoeae*. No statistically significant differences in prevalence were observed between pregnant and non-pregnant women for any pathogen. **Conclusions**: The prevalence of *M. genitalium* in this Japanese cohort was comparable to that of *C. trachomatis* in both pregnant and non-pregnant women, highlighting its significance as a major STI pathogen. These findings underscore the importance of including *M. genitalium* in routine STI screening panels for symptomatic women and antenatal care to prevent reproductive health complications. Given the high rates of antimicrobial resistance documented in Japanese *M. genitalium* strains, specific diagnostic testing is essential to enable targeted, resistance-guided therapy.

## 1. Introduction

*Mycoplasma genitalium* has emerged as a significant pathogen responsible for sexually transmitted infections (STIs) globally. Since its first isolation from men with non-gonococcal urethritis (NGU) in the early 1980s, it has been firmly established as a causative agent of NGU in men. It is increasingly associated with cervicitis, pelvic inflammatory disease (PID), preterm birth, and tubal factor infertility in women [1,2]. Despite its clinical importance, *M. genitalium* is not routinely included in STI screening panels in many countries, including Japan, leading to potential underdiagnosis and untreated infections that may result in serious reproductive health complications.

The global epidemiology of *M. genitalium* has been extensively documented in recent systematic reviews and meta-analyses. Baumann et al. [3] reported an estimated global prevalence of 1.3% in general populations, with substantially higher rates of 3.9% among attendees of sexual health clinics, 5.2% in men who have sex with men, and 6.9% in female sex workers. Regional variations are notable, with prevalence rates ranging from 1% to 6% reported in the general populations across North America, Europe, and parts of Asia [3,4]. In pregnancy, international data suggest prevalence rates of approximately 1% in high-income settings, though substantially higher rates have been documented in resource-limited settings, including 12.6% in Zambia and 3.6% to 12.5% in Papua New Guinea and South Africa [5,6,7]. These geographic and population-specific variations underscore the importance of local epidemiological data for informing clinical practice and public health policy.

In Japan, the epidemiology of *M. genitalium* has been predominantly characterized in male populations, with limited data available for women. Multiple studies have documented that *M. genitalium* accounts for 10% to 20% of non-gonococcal urethritis cases in Japanese men, with detection rates ranging from 13% to 20% in symptomatic male patients [8,9]. A comprehensive review by Deguchi et al. [8] synthesized Japanese data showing consistent detection of *M. genitalium* in 10% to 20% of men with NGU across multiple centers. Furthermore, Japan has been recognized as an endemic area for antimicrobial-resistant *M. genitalium*, with recent surveillance in Tokyo documenting alarmingly high rates of macrolide resistance approaching 90% and increasing fluoroquinolone resistance [10,11]. These resistance patterns have significant implications for treatment strategies and highlight the urgent need for diagnostic testing to enable resistance-guided therapy.

In stark contrast to the robust data available for men, epidemiological information on *M. genitalium* in Japanese women remains remarkably limited. One of the earliest studies, conducted by Uno et al. [12], examined 200 Japanese women and detected *M. genitalium* in 7.8% of women with cervicitis and 5.7% with adnexitis, but found no infections among 80 asymptomatic pregnant women. Subsequent studies have been scarce, creating a substantial knowledge gap regarding the contemporary prevalence and epidemiological characteristics of *M. genitalium* infection in Japanese women, particularly in the context of routine gynecological care and antenatal screening. This paucity of data is particularly concerning given the documented associations between *M. genitalium* and adverse reproductive outcomes in women, including cervicitis, endometritis, PID, and potentially preterm birth and spontaneous abortion [1,2,13,14].

While *Chlamydia trachomatis* and *Neisseria gonorrhoeae* have well-established national surveillance systems in Japan, systematic data collection for *M. genitalium* has not been implemented. Furthermore, diagnostic testing for *M. genitalium* is not covered by the Japanese national health insurance system, which creates significant barriers to both clinical diagnosis and epidemiological surveillance [8]. This lack of reimbursement has resulted in limited clinical testing, contributing to the sparse epidemiological data available for Japanese women. Understanding local prevalence is critical for guiding empirical treatment strategies, informing screening recommendations, and advocating for policy changes regarding diagnostic test coverage, especially given the rising concern of antimicrobial resistance in *M. genitalium* strains circulating in Japan.

The clinical significance of *M. genitalium* extends beyond acute infection. In women, untreated or inadequately treated *M. genitalium* infections can lead to ascending infection of the upper reproductive tract, resulting in endometritis and PID [1]. These conditions are associated with significant long-term sequelae, including chronic pelvic pain, ectopic pregnancy, and tubal factor infertility [2]. Moreover, emerging evidence suggests that *M. genitalium* infection during pregnancy may be associated with adverse outcomes such as preterm birth, premature rupture of membranes, and low birth weight, although the strength of these associations varies across studies [5,13,14]. Given these potential complications, understanding the prevalence of *M. genitalium* in both pregnant and non-pregnant women is essential for developing appropriate screening and management strategies.

This study aims to address the critical knowledge gap regarding *M. genitalium* epidemiology in Japanese women by investigating the prevalence of *M. genitalium*, *C. trachomatis*, and *N. gonorrhoeae* in a cohort of both pregnant and non-pregnant women attending gynecological clinics in Gifu, Japan. We further analyze the rates of co-infection among these pathogens to provide contemporary epidemiological data that can inform clinical practice, guide treatment decisions, and contribute to the development of evidence-based public health policy for STI screening and management in Japan. By comparing prevalence rates between pregnant and non-pregnant women, we also seek to determine whether *M. genitalium* screening should be incorporated into routine antenatal care protocols alongside the currently recommended screening for *C. trachomatis* and *N. gonorrhoeae*.

## 2. Materials and Methods

### 2.1. Study Setting and Population

This retrospective cross-sectional study analyzed data derived from vaginal swabs submitted for STI testing between April 2021 and November 2022. The specimens were obtained from patients visiting the Ai Ladies Clinic and the Takahashi Ladies Clinic, both located in Gifu City, Gifu Prefecture, Japan. These clinics provide general gynecological services, including STI screening, contraceptive counseling, infertility, and antenatal care to the local community. In this retrospective laboratory-based dataset, only limited clinical background information was consistently available. Detailed individual-level demographic variables, such as age distributions, were not uniformly retrievable for all participants.

The study population was categorized into two groups: non-pregnant women (n = 2138) and pregnant women (n = 236). Non-pregnant patients typically presented for consultation due to urogenital symptoms, including urethral symptoms (dysuria or urethral discharge), vaginal symptoms (abnormal vaginal discharge, dysuria, or abnormal bleeding), lower abdominal pain, or as part of contact tracing following notification of a sexual partner’s STI diagnosis. Some asymptomatic women requesting routine STI screening were also included. Pregnant women were screened as part of routine antenatal care procedures, typically during their first prenatal visit, regardless of symptom status. Accordingly, the non-pregnant cohort should be interpreted primarily as a clinically indicated testing population rather than a general screening population. All participants who underwent STI testing during the study period and had valid test results for all three pathogens (*M. genitalium*, *C. trachomatis,* and *N. gonorrhoeae*) were included in the analysis.

### 2.2. Sampling and Molecular Testing

Vaginal swab specimens were collected by trained clinicians using standardized collection techniques. Each specimen was obtained using a sterile swab inserted into the vaginal canal and rotated against the vaginal wall to ensure adequate cellular material collection. The swabs were immediately transferred to the appropriate transport media provided by the manufacturer. All samples were transported to the testing laboratory on the same day as collection at ambient temperature to maintain specimen integrity.

Molecular detection was performed using nucleic acid amplification tests (NAATs), which are considered the gold standard for STI diagnosis due to their high sensitivity and specificity. For the detection of *M. genitalium*, *C. trachomatis*, and *N. gonorrhoeae*, the cobas^®^ CT/NG and cobas^®^ TV/MG assays (Roche Diagnostics, Basel, Switzerland) were utilized. These assays are qualitative in vitro diagnostic tests that employ real-time polymerase chain reaction (PCR) technology to detect specific DNA target regions of the respective pathogens. The cobas^®^ CT/NG assay simultaneously detects *C. trachomatis* and *N. gonorrhoeae*, while the cobas^®^ TV/MG assay simultaneously detects *Trichomonas vaginalis* and *M. genitalium*. All assays were performed on the automated cobas^®^ 4800 system (Roche Diagnostics, Basel, Switzerland) according to the manufacturer’s instructions, with appropriate positive and negative controls included in each run to ensure assay validity.

It should be noted that antimicrobial resistance testing for *M. genitalium* was not performed in this study, as the primary objective was to determine prevalence rates rather than characterize resistance patterns. However, given the high rates of macrolide and fluoroquinolone resistance documented in Japanese *M. genitalium* strains, this represents an important limitation of our study.

### 2.3. Statistical Analysis

Statistical analyses were conducted using RStudio (version 2022.07.1; RStudio, PBC, Boston, MA, USA) with R version 4.2.1 (R Foundation for Statistical Computing, Vienna, Austria). Prevalence rates for each pathogen were calculated as the proportion of positive cases divided by the total number of specimens tested, expressed as percentages with exact 95% confidence intervals (CIs). The exact 95% CIs for prevalence estimates were calculated using the Clopper-Pearson method for binomial proportions, which is particularly appropriate for studies with relatively small numbers of positive cases.

Co-infection rates were determined by identifying specimens that were simultaneously positive for multiple pathogens. The proportions of co-infections among all tested specimens and among pathogen-positive cases were calculated with 95% CIs. To compare prevalence rates between pregnant and non-pregnant women, Fisher’s exact test was employed due to the relatively small number of positive cases in some categories. A two-sided *p*-value of less than 0.05 was considered statistically significant.

Descriptive statistics were used to summarize the demographic and clinical characteristics of the study population. As this was primarily a descriptive epidemiological study focused on prevalence estimation, formal hypothesis testing was limited to comparisons between the two population groups (pregnant versus non-pregnant women). All statistical analyses were performed in accordance with standard epidemiological methods for cross-sectional studies.

### 2.4. Ethical Considerations

This retrospective study was conducted in accordance with the Declaration of Helsinki and approved by the Ethics Committee of Aichi Medical University (ethic code [2021-038], approved on 7 June 2021). Despite the retrospective nature of this study using de-identified laboratory data collected during routine clinical practice, ethical approval was obtained from the institutional review board, and written informed consent was obtained from all participants. All data were anonymized prior to analysis, and patient confidentiality was strictly maintained throughout the study.

## 3. Results

### 3.1. Study Population Characteristics

A total of 2374 women were included in this cross-sectional study, comprising 2138 non-pregnant women and 236 pregnant women who underwent STI testing between April 2021 and November 2022. All participants had complete test results for *M. genitalium*, *C. trachomatis,* and *N. gonorrhoeae*. The non-pregnant cohort primarily consisted of women presenting with urogenital symptoms or identified through sexual partner contact tracing, while the pregnant cohort represented women undergoing routine antenatal screening. Because this was a retrospective laboratory-based analysis, quantitative individual-level summaries of age and symptom categories were not consistently available for all participants.

### 3.2. Prevalence of STI Pathogens in Non-Pregnant Women

Among the 2138 non-pregnant women analyzed, the overall positivity rate for *M. genitalium* was 3.8% (82/2138; 95% CI: 3.1–4.7%), which was slightly higher than that of *C. trachomatis* at 3.4% (72/2138; 95% CI: 2.7–4.2%). *N. gonorrhoeae* was the least common pathogen, detected in only 0.4% (9/2138; 95% CI: 0.2–0.8%) of the women. The comparable prevalence rates of *M. genitalium* and *C. trachomatis* highlight the clinical significance of *M. genitalium* as a major STI pathogen in this population.

Regarding mono-infections, *M. genitalium* was detected as a single pathogen in 3.5% (76/2138; 95% CI: 2.8–4.4%) of cases, while *C. trachomatis* mono-infection was found in 3.1% (66/2138; 95% CI: 2.4–3.9%). *N. gonorrhoeae* mono-infection was detected in only 0.2% (4/2138; 95% CI: 0.05–0.5%) of cases.

Co-infections were relatively rare in this cohort. Simultaneous infection with *C. trachomatis* and *M. genitalium* occurred in 0.2% (6/2138; 95% CI: 0.09–0.5%) of patients, representing 7.3% (6/82) of *M. genitalium*-positive cases and 8.3% (6/72) of *C. trachomatis*-positive cases. Co-infection with *C. trachomatis* and *N. gonorrhoeae* was observed in 0.2% (5/2138; 95% CI: 0.08–0.5%) of the cohort. Notably, there were no cases of co-infection involving *M. genitalium* and *N. gonorrhoeae* without *C. trachomatis*, and no triple infections (all three pathogens simultaneously) were identified. Detailed distributions of infection patterns are presented in Table 1.

### 3.3. Prevalence of STI Pathogens in Pregnant Women

Among the 236 pregnant women screened during routine antenatal care, the prevalence patterns were remarkably similar to those observed in the non-pregnant group. *M. genitalium* and *C. trachomatis* both showed an overall positivity rate of 3.8% (9/236; 95% CI: 1.8–7.1%), while *N. gonorrhoeae* was detected in only one case (0.4%; 95% CI: 0.01–2.3%).

Single infections of *M. genitalium* and *C. trachomatis* were both identified in 3.4% (8/236; 95% CI: 1.5–6.5%) of the pregnant cohort. *N. gonorrhoeae* mono-infection was detected in one case (0.4%; 95% CI: 0.01–2.3%). Only one case of co-infection (*C. trachomatis* and *M. genitalium*) was identified, representing 0.4% (1/236; 95% CI: 0.01–2.3%) of the pregnant population. This co-infection accounted for 11.1% (1/9) of *M. genitalium*-positive cases and 11.1% (1/9) of *C. trachomatis*-positive cases among pregnant women. No other co-infection combinations were observed, and no triple infections were detected. Detailed distributions of infection patterns in pregnant women are presented in Table 2.

### 3.4. Comparison Between Pregnant and Non-Pregnant Women

Comparative analysis between pregnant and non-pregnant women revealed no statistically significant differences in the prevalence of any of the three pathogens examined. For *M. genitalium*, the prevalence was identical in both groups (3.8%; *p* = 1.000, Fisher’s exact test). Similarly, no significant difference was observed for *C. trachomatis* (3.4% in non-pregnant versus 3.8% in pregnant women; *p* = 0.744) or *N. gonorrhoeae* (0.4% in both groups; *p* = 1.000). These findings suggest that pregnancy status does not significantly influence the prevalence of these STI pathogens in women attending gynecological clinics in this region. However, the relatively small sample size of pregnant women limits the statistical power to detect modest differences.

The co-infection rate between *M. genitalium* and *C. trachomatis* was also similar between the two groups (0.2% in non-pregnant versus 0.4% in pregnant women; *p* = 0.527), indicating that co-infection patterns do not differ substantially by pregnancy status.

## 4. Discussion

This study provides contemporary data on the prevalence of *M. genitalium* in Japanese women, revealing a positivity rate of 3.8% in both pregnant and non-pregnant women attending gynecological clinics in Gifu, Japan. These findings are particularly significant as they demonstrate that *M. genitalium* prevalence is comparable to that of *C. trachomatis* (3.4% in non-pregnant and 3.8% in pregnant women) in this population, highlighting *M. genitalium* as a major STI pathogen that warrants greater clinical attention. However, given the single-region clinic-based design of this study, our findings should be interpreted as supportive evidence for further evaluation of targeted screening strategies rather than as definitive evidence for universal routine screening.

Our results align with recent international studies indicating that *M. genitalium* prevalence often rivals that of *C. trachomatis* in certain symptomatic populations and clinical settings. A systematic review and meta-analysis by Baumann et al. [3] reported global *M. genitalium* prevalence estimates of 1.3% in general populations, with higher rates of 3.9% among attendees of sexual health clinics and substantially elevated rates in high-risk groups. The prevalence of 3.8% in our non-pregnant cohort—primarily comprising women with urogenital symptoms or contact history—is consistent with recent literature from Asia and other regions. A 2024 study among Vietnamese participants in an HIV pre-exposure prophylaxis program reported high *M. genitalium* prevalence rates [4], while studies from other Asian regions have documented prevalences ranging from 3% to 13% depending on population characteristics and testing methodologies [15]. The consistency of our findings with international data supports the validity of our results and suggests that *M. genitalium* is a common STI pathogen in Japanese women, comparable to other well-established pathogens.

The similarity in infection rates between pregnant and non-pregnant women in our study (3.8% in both groups) is particularly noteworthy and has important implications for antenatal care. While pregnant women are routinely screened for *C. trachomatis* and *N. gonorrhoeae* as part of standard antenatal care in Japan, *M. genitalium* is not included in standard screening protocols. Recent evidence has strengthened the association between *M. genitalium* and adverse pregnancy outcomes. A 2022 systematic review and meta-analysis by Frenzer et al. [13] found that *M. genitalium* infection may be associated with an increased risk of preterm birth, although the authors noted heterogeneity across studies. Additionally, a 2022 study by Jonduo et al. [14] demonstrated associations between genital mycoplasmas and various adverse pregnancy outcomes, though the authors acknowledged limitations in drawing definitive conclusions due to confounding factors. More recently, a 2025 study from Zambia by Schröder et al. [5] reported a 12.6% prevalence of *M. genitalium* among pregnant women, emphasizing the potential for adverse pregnancy outcomes and the importance of appropriate screening and treatment. A 2024 study by Scoullar et al. [16] examining *M. genitalium* in pregnancy specifically addressed co-infections with other STIs, noting that 18–35% of *M. genitalium*-positive individuals had concurrent STIs, with *C. trachomatis* being the most common. These collective findings support the consideration of broader screening protocols in antenatal care, particularly for populations with symptoms, risk factors, or in regions with high prevalence rates.

The co-infection rate between *M. genitalium* and *C. trachomatis* was relatively low in our study (0.2% in non-pregnant women and 0.4% in pregnant women, representing 7.3% and 11.1% of *M. genitalium*-positive cases, respectively). This observation suggests that while these infections share similar transmission routes and risk factors, they may be circulating somewhat independently within this community rather than strictly clustering in the same individuals. However, the clinical significance of even low rates of co-infection should not be underestimated, as co-infected individuals may require modified treatment strategies to address both pathogens effectively. The distinction between mono-infection and co-infection is important for clinical management and treatment selection. Notably, 55.6% (5/9) of *N. gonorrhoeae*-positive cases in the non-pregnant cohort occurred as co-infections with *C. trachomatis*, a pattern that is broadly consistent with reports from other clinical cohorts worldwide.

The clinical implications of our findings are substantial for Japanese healthcare practice. Currently, *M. genitalium* testing is not covered by the Japanese national health insurance system, creating significant barriers to both clinical diagnosis and epidemiological surveillance [8]. Given that *M. genitalium* prevalence in our study was comparable to that of *C. trachomatis*, which is routinely screened and reimbursed, our results support consideration of incorporating *M. genitalium* into targeted STI testing strategies, particularly for symptomatic women and selected antenatal care settings where local prevalence, clinical risk, and cost-effectiveness justify such an approach. The potential cost-effectiveness of such screening should be evaluated in future studies, considering the documented associations between *M. genitalium* infection and adverse reproductive outcomes, including cervicitis, pelvic inflammatory disease, preterm birth, and potential long-term sequelae such as tubal factor infertility [1,2,13]. Economic analyses incorporating direct medical costs, indirect costs related to complications, and quality-adjusted life years would provide valuable data to inform policy decisions regarding insurance coverage for *M. genitalium* testing.

Furthermore, the therapeutic implications of our findings cannot be overstated in the context of Japan’s alarming antimicrobial resistance rates. Current international guidelines, including those from the Centers for Disease Control and Prevention (CDC) and European guidelines, do not recommend routine screening for asymptomatic *M. genitalium* infection, but emphasize that testing should be considered in symptomatic patients, particularly when initial treatment for urethritis or cervicitis fails [17,18]. However, the syndromic management approach of treating urogenital symptoms with empirical azithromycin monotherapy—a common strategy for presumed chlamydial infections—may be inadequate for patients with *M. genitalium* infection. Recent surveillance data from Tokyo have documented macrolide resistance approaching 90% and rising fluoroquinolone resistance in *M. genitalium* isolates [10,11]. A comprehensive 2025 systematic review by Chua et al. [19] documented evolving patterns of macrolide and fluoroquinolone resistance in *M. genitalium* worldwide, with Japan identified as a region of particular concern. Most recently, a 2025 study by Kikuchi et al. [10] found a high prevalence of macrolide resistance-associated mutations and emerging fluoroquinolone resistance, with dual-resistant strains spreading in certain populations in Tokyo. This alarming resistance profile underscores the critical need for specific *M. genitalium* diagnostic testing followed by resistance-guided therapy to prevent treatment failures, reduce complications, and limit further transmission of resistant strains. The use of empirical azithromycin without documented *M. genitalium* testing may inadvertently contribute to the selection and spread of macrolide-resistant strains.

The issue of antimicrobial resistance in *M. genitalium* has become increasingly concerning globally and represents one of the most pressing challenges in STI management. A comprehensive 2025 systematic review by Chua et al. [19] documented evolving patterns of macrolide and fluoroquinolone resistance worldwide, with some regions experiencing dual-class resistance exceeding 10%. In Japan specifically, the situation is particularly dire. The work by Jensen and Unemo [20] highlighted that antimicrobial treatment and resistance in sexually transmitted bacterial infections, including *M. genitalium*, represent a growing threat to public health. The high resistance rates documented in Japan suggest that treatment protocols need urgent revision, with consideration of first-line therapies such as extended-course moxifloxacin (for quinolone-susceptible strains) or newer agents such as pristinamycin or lefamulin, where available. Ideally, resistance testing should be performed prior to treatment initiation, although practical and economic barriers currently limit widespread implementation of this approach in Japan.

This study has several important limitations that should be acknowledged. First, the relatively small sample size of pregnant women (n = 236) compared to the non-pregnant group (n = 2138) limits the precision of prevalence estimates in the obstetric population and reduces statistical power to detect modest differences between groups. The wide confidence intervals for prevalence estimates in pregnant women reflect this limitation. Second, as a laboratory-based retrospective study, detailed clinical correlates such as specific symptom profiles, symptom duration and severity, comprehensive sexual behavior history (including number of sexual partners, condom use, and history of previous STIs), and pregnancy outcomes were not available for individual-level analysis. In particular, quantitative age-stratified summaries and standardized symptom-status data were not consistently retrievable for all participants from the available dataset. Such information would have provided valuable insights into risk factors and clinical presentations associated with *M. genitalium* infection. Third, and most importantly, antimicrobial resistance testing was not performed in this dataset. Given the documented high rates of macrolide and fluoroquinolone resistance in Japan, future prospective studies should incorporate molecular resistance testing (detection of mutations in the 23S rRNA gene for macrolide resistance and *par*C gene for quinolone resistance) to guide appropriate treatment strategies and monitor resistance trends over time.

Additionally, our study population was derived exclusively from two gynecological clinics in Gifu city, which may limit the generalizability of our findings to other geographic regions of Japan or to populations accessing care through different healthcare settings such as public health centers, university hospitals, or specialized STI clinics. The prevalence estimates may not be representative of asymptomatic women in the general population, as the non-pregnant cohort primarily consisted of symptomatic patients or those identified through contact tracing. The pregnant cohort, while screened as part of routine antenatal care, may not represent the general pregnant population, as women who access prenatal care at private clinics may differ in socioeconomic status and health-seeking behaviors from those accessing care through public facilities. Finally, we did not collect detailed information on sexual behaviors, number of sexual partners, history of previous STIs, or other important risk factors that could have provided additional epidemiological insights and enabled risk stratification analyses. Future studies should incorporate comprehensive questionnaires to identify specific risk factors for *M. genitalium* infection among Japanese women and to characterize high-risk populations that may benefit most from targeted screening.

Despite these limitations, our study provides valuable contemporary data on *M. genitalium* prevalence in a substantial cohort of Japanese women and contributes important epidemiological information to the limited existing literature. The use of highly sensitive and specific nucleic acid amplification tests (Aptima™ TMA assays) enhances the reliability of our prevalence estimates. The inclusion of both pregnant and non-pregnant women allows for comparative analysis and provides data relevant to different clinical settings and screening scenarios.

In conclusion, *M. genitalium* was identified in 3.8% of both pregnant and non-pregnant women in this Japanese cohort from Gifu, a prevalence comparable to that of *C. trachomatis* and substantially higher than that of *N. gonorrhoeae*. The significant presence of *M. genitalium* warrants increased clinical awareness and incorporation of diagnostic testing into clinical algorithms for women presenting with urogenital symptoms. Our findings also support further investigation of whether selected antenatal care populations may benefit from expanded testing, rather than establishing a recommendation for universal routine screening on the basis of this study alone. Given the high antimicrobial resistance rates documented in Japanese *M. genitalium* strains, specific diagnostic testing is essential to enable targeted, resistance-guided therapy and prevent treatment failures that could lead to persistent infection, ongoing transmission, and serious reproductive health complications. Future research should focus on characterizing antimicrobial resistance patterns in *M. genitalium* isolates from Japanese women, evaluating the cost-effectiveness of expanded screening programs, investigating clinical outcomes and pregnancy complications associated with *M. genitalium* infection, and assessing optimal treatment strategies in the context of high-level antimicrobial resistance. Policy initiatives to include *M. genitalium* testing in national health insurance coverage should be pursued, given the growing evidence of its clinical significance and public health impact.

## Figures and Tables

**Table 1:** Prevalence of urogenital pathogens among non-pregnant women (n = 2138).

Infection Status	Count (n)	Percentage (%)	95% CI
Mono-infections			
*M. genitalium* only	76	3.5	2.8–4.4
*C. trachomatis* only	66	3.1	2.4–3.9
*N. gonorrhoeae* only	4	0.2	0.05–0.5
Co-infections			
*C. trachomatis* + *M. genitalium*	6	0.2	0.09–0.5
*C. trachomatis* + *N. gonorrhoeae*	5	0.2	0.08–0.5
*M. genitalium* + *N. gonorrhoeae*	0	0.0	0.0–0.2
Triple infection (CT + NG + MG)	0	0.0	0.0–0.2
Total Positivity (Overall)			
*M. genitalium* Total	82	3.8	3.1–4.7
*C. trachomatis* Total	72	3.4	2.7–4.2
*N. gonorrhoeae* Total	9	0.4	0.2–0.8

CI, confidence interval; CT, *Chlamydia trachomatis*; MG, *Mycoplasma genitalium*; NG, *Neisseria gonorrhoeae*.

**Table 2:** Prevalence of urogenital pathogens among pregnant women (n = 236).

Infection Status	Count (n)	Percentage (%)	95% CI
Mono-infections			
*M. genitalium* only	8	3.4	1.5–6.5
*C. trachomatis* only	8	3.4	1.5–6.5
*N. gonorrhoeae* only	1	0.4	0.01–2.3
Co-infections			
*C. trachomatis* + *M. genitalium*	1	0.4	0.01–2.3
*C. trachomatis* + *N. gonorrhoeae*	0	0.0	0.0–1.5
*M. genitalium* + *N. gonorrhoeae*	0	0.0	0.0–1.5
Triple infection (CT + NG + MG)	0	0.0	0.0–1.5
Total Positivity (Overall)			
*M. genitalium* Total	9	3.8	1.8–7.1
*C. trachomatis* Total	9	3.8	1.8–7.1
*N. gonorrhoeae* Total	1	0.4	0.01–2.3

CI, confidence interval; CT, *Chlamydia trachomatis*; MG, *Mycoplasma genitalium*; NG, *Neisseria gonorrhoeae*.

## Data Availability

The data presented in this study are available on request from the corresponding author due to privacy and ethical restrictions concerning patient information.

## Abbreviations

The following abbreviations are used in this manuscript:

STI	Sexually Transmitted Infections
NGU	Non-Gonococcal Urethritis
PID	Pelvic Inflammatory Disease
NAATs	Nucleic Acid Amplification Tests
PCR	Polymerase Chain Reaction
CI	confidence interval
CT	*Chlamydia trachomatis*
MG	*Mycoplasma genitalium*
NG	*Neisseria gonorrhoeae*
